# Impact of Ferrous Iron on Microbial Community of the Biofilm in Microbial Fuel Cells

**DOI:** 10.3389/fmicb.2017.00920

**Published:** 2017-06-07

**Authors:** Qian Liu, Bingfeng Liu, Wei Li, Xin Zhao, Wenjing Zuo, Defeng Xing

**Affiliations:** State Key Laboratory of Urban Water Resource and Environment, School of Municipal and Environmental Engineering, Harbin Institute of TechnologyHarbin, China

**Keywords:** microbial fuel cell, ferrous iron, electricity generation, microbial community, high throughput sequencing

## Abstract

The performance of microbial electrochemical cells depends upon microbial community structure and metabolic activity of the electrode biofilms. Iron as a signal affects biofilm development and enrichment of exoelectrogenic bacteria. In this study, the effect of ferrous iron on microbial communities of the electrode biofilms in microbial fuel cells (MFCs) was investigated. Voltage production showed that ferrous iron of 100 μM facilitated MFC start-up compared to 150 μM, 200 μM, and without supplement of ferrous iron. However, higher concentration of ferrous iron had an inhibitive influence on current generation after 30 days of operation. Illumina Hiseq sequencing of 16S rRNA gene amplicons indicated that ferrous iron substantially changed microbial community structures of both anode and cathode biofilms. Principal component analysis showed that the response of microbial communities of the anode biofilms to higher concentration of ferrous iron was more sensitive. The majority of predominant populations of the anode biofilms in MFCs belonged to *Geobacter*, which was different from the populations of the cathode biofilms. An obvious shift of community structures of the cathode biofilms occurred after ferrous iron addition. This study implied that ferrous iron influenced the power output and microbial community of MFCs.

## Introduction

Microbial electrochemical cell (MEC) has been admired as a versatile device that can be used for alternative energy generation, electrosynthesis, biosensor, and waste treatment (Hou et al., 2016; Liu et al., 2016a; Huang et al., 2017). However, practical implementation of microbial fuel cells (MFCs) remains restricted by reasons of low electron transfer efficiency and high material costs (Logan et al., 2006). For the past few years, researchers studied electrode materials, exoelectrogenic bacteria, reactor configuration and operational conditions of MFCs (Watson and Logan, 2010; Yong et al., 2011; Janicek, 2015), and pointed out that microbial biofilm was the most direct and key element that affect current generation (Mohan et al., 2008). However, microbial biofilm and its community structure of MFCs can be influenced by temperature, pH, carbon source, inoculum, and metal ion (Lu et al., 2011, 2012; Patil et al., 2011; Wu et al., 2013). The diverse populations developed in the biofilms in MECs have been widely analyzed (Mei et al., 2015). *Geobacter* as a typical dissimilatory metal-reducing bacterium (DMRB) is commonly identified in MFCs (Mohan et al., 2014; Zhu et al., 2014; Kumar et al., 2016). Hence, to understand and optimize ecological conditions that facilitate exoelectrogens enrichment and electron transfer are essential for MEC application.

Iron plays a central role in the development and maintenance of biofilm of *Pseudomonas* (Hunter et al., 2013). Although ferric iron has been identified as an important parameter affecting the biofilm formation (Banin et al., 2005), the impact of ferrous iron on the biofilm is less known. Metal ions are essential minerals to composite microorganisms and biological molecules, including metalloproteins which play key roles in most biological processes (iron for respiration; Cvetkovic et al., 2010). The reactive metal ions may have the phenomenon of redox reaction, catalysis, or precipitation, etc. and thus directly affect the performance of MECs by influencing the metabolism of microorganisms or the activity of enzymes (Lu et al., 2015). Due to its high redox activity, the Fe^2+^ is able to be oxidized at the anode in an air-cathode fuel cells which are capable of abiotic electricity generation (Cheng et al., 2007). The addition of ferrous sulfate to the anode medium has improved the power densities of MFCs during start-up period (Wei et al., 2013). However, there are less literatures concerning the response of exoelectrogenic community in the electrode biofilms to ferrous iron.

Ferrous iron used in catholyte of dual-chambered MFC enhanced power output by increasing salt concentration or improving cathode potential (Ter Heijne et al., 2007). A comparison of results with and without ferrous iron as a cathodic reactant also revealed that the addition of ferrous iron enhanced power generation in batch MFC (Wang et al., 2011). However, the knowledge related to the effects of ferrous iron on performances of MFCs and microbial communities of electrode biofilms is less known. To reveal the response of microbial community of the electrode biofilm to ferrous iron, in this study, electrochemical performances of MFCs supplemented with different concentrations of ferrous iron were investigated. Meanwhile, microbial community structures of the anodes and cathodes biofilms in MFCs were analyzed using Illumina Hiseq sequencing of 16S rRNA gene amplicons.

## Materials and Methods

### MFC Configuration and Operation

Single-chamber MFCs with volume of 14 mL were constructed as previously described (Xing et al., 2008). Anodes were made of carbon paper (Toray TGP-H-090, Japan), while cathodes were stainless steel mesh by rolling activated carbon and polytetrafluoroethylene (PTFE) (Dong et al., 2012) (the area of anode and cathode were both 7 cm^2^). Domestic wastewater was used as inoculum in the first 5 days. Nutrient solutions were consisted of 1 g/L sodium acetate, 5 mL/L vitamins, 12.5 mL/L minerals, 100 mM phosphate buffer saline (PBS, pH of 6) and FeSO_4_ with different concentrations. The final pH value of nutrient solution was 6.2 ± 0.1. The final concentrations of FeSO_4_ in MFCs were 32 (control), 100, 150, and 200 μM.

Voltages across the external resistor (1000 Ω) of MFCs were measured using Keithley 2700 multimeter/data acquisition system. All MFCs were operated at 35°C and each Fe^2+^ concentration have three replicates. Cyclic voltammetry (CV) measurements of MFCs at the 15th day were performed on Autolab potentiostat (Metrohm, Netherlands) with scan rate of 0.01 V/s.

### DNA Extraction and Illumina Sequencing of 16S rRNA Gene

After MFCs were operated for 2 months, the anode and cathode biofilms of MFCs (control, fed with 100 and 200 μM Fe^2+^) were sampled for genomic DNA extraction by using PowerSoil DNA Isolation Kit according the manufacturer’s instructions. DNA concentration and purity were determined by NanoPhotometer P-Class (Implen, GmbH). Prior to polymerase chain reaction (PCR) amplification, DNA of anode and cathode biofilms from three duplicated bioreactors were mixed. The V4 region (length of ∼373 bp) of bacterial 16S rRNA gene was amplified by using a set of bacterial primers 515F (5′-GTGCCAGCMGCCGCGGTAA-3′) and 806R (5′-GGACTACHVGGGTWTCTAAT-3′). After integrated with barcode, PCR amplification was implemented by using ABI GeneAmp^®^ 9700 PCR system.

Sequencing was performed on Illumina Hiseq platforms according to the standard protocols. Raw Tags were overlapped by using the Fast Length Adjustment of SHort reads (FLASH; V1.2.7)^1^ software (Magoc and Salzberg, 2011) and filtered following pipelines of Quantitative Insights Into Microbial Ecology (QIIME, V1.7.0; Caporaso et al., 2010). Effective tags were obtained by removing chimeric sequences after aligned using Gold database^2^. Operational taxonomic units (OTUs) were determined based on the threshold of 97% similarity using UPARSE software (Uparse V7.0.1001). A representative sequence of each OTU was aligned for taxonomic identification using the GreenGene database^3^ and Ribosomal Database Project (RDP) classifier (version 2.2)^4^ with the threshold of 80–100% (DeSantis et al., 2006; Wang et al., 2007). The raw Illumina sequencing data were deposited in the Sequence Read Archive (SRA) of National Center for Biotechnology Information (NCBI) under the accession Nos. SRR5266191–SRR5266196.

## Results and Discussion

### Electricity Generation and Electrochemical Activity of MFCs

**FIGURE 1:**
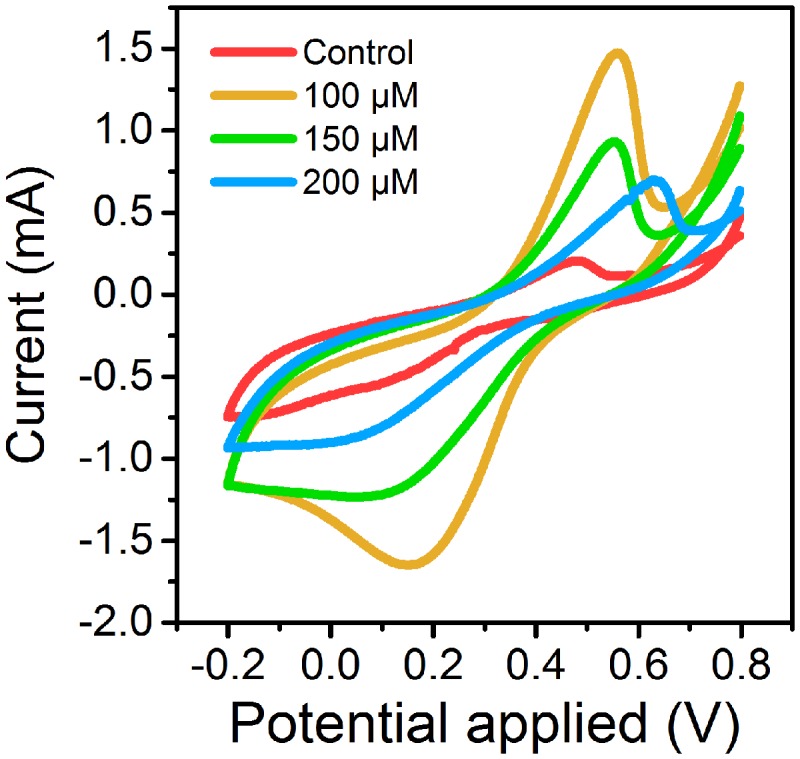
**Cyclic voltammetry curves of MFCs supplemented with different concentrations of ferrous iron on 15th day**.

**FIGURE 2:**
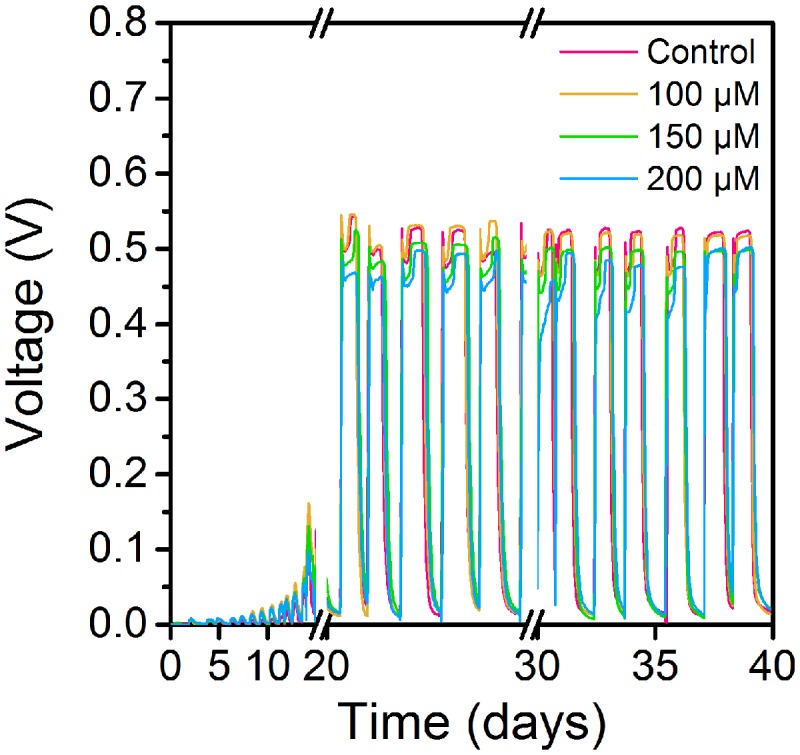
**Voltage curves of MFCs supplemented with ferrous iron of different concentrations**.

Cyclic voltammetry curves showed that MFCs supplemented with 100 μM ferrous ion (Fe^2+^) obtained the highest current peak on the 15th day (**Figure 1**). The results suggested that low concentration of Fe^2+^ could obviously improve electrochemical activity of MFCs in the start-up period. During another 15 days of operation, MFCs with 100 μM ferrous ion showed the best electrochemical characteristics compared to MFCs with 150 and 200 μM Fe^2+^, and MFCs without additional Fe^2+^ supplement (**Figure 2**). The maximum voltage of 0.55 V was monitored in MFCs fed with 100 μM Fe^2+^, and then following the order control (0.54 V), 150 μM Fe^2+^ (0.52 V) and 200 μM Fe^2+^ (0.47 V). After all MFCs were operated for 30 days, MFCs of control groups maintained the steady voltage output, while other MFCs with Fe^2+^ addition performed a weaken efficiency.

### Community Diversity of MFCs with Different Concentrations of Fe^2+^

**FIGURE 3:**
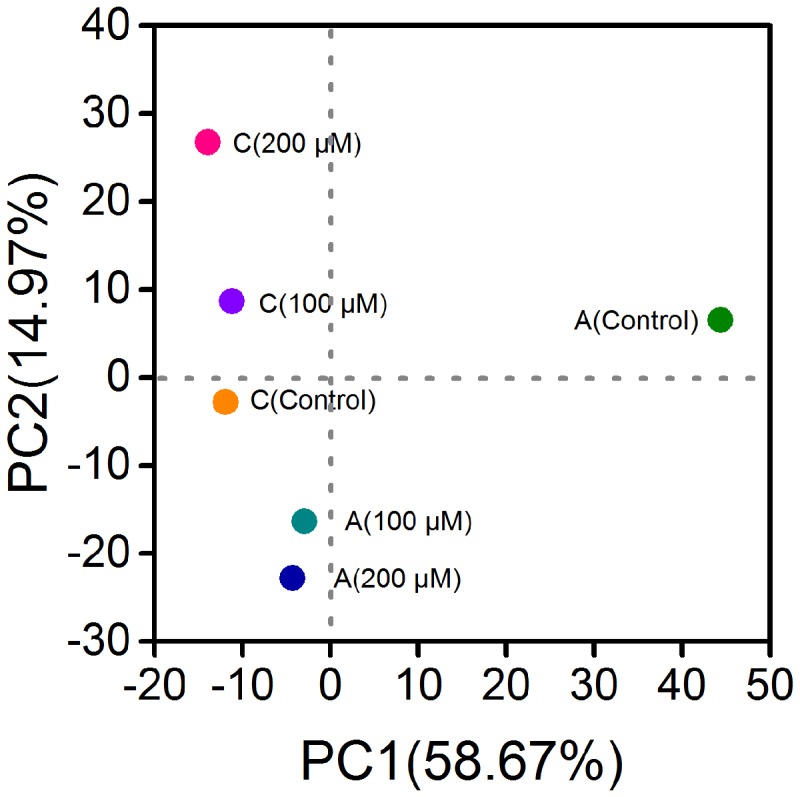
**Principal component analysis based on operational taxonomic units of the anode and cathode biofilms of MFCs**.

Since the power outputs of MFCs with 150 and 200 μM were similar, and the CV result of 200 μM adequately represented the decrease of electrochemical activity of electrode biofilms, the biofilm samples of MFCs with ferrous iron of 150 μM were not used for microbial community analysis. After quality filtering the raw tags, 50,373 to 54,932 effective tags were obtained per sample, with average length of 373 bp. Total OTUs at the 97% similarity were ranged from 630 to 824 per sample with an average of 710 OTUs (**Table 1**). The anode biofilms in MFCs supplemented with ferrous iron showed slightly lower population diversity than that in control MFCs without ferrous iron supplement. Shannon indices were 3.72, 4.71, and 5.21 for the anodes biofilms with 100, 200 μM Fe^2+^, and without Fe^2+^, respectively. By contrast, Fe^2+^ increased the population diversities of the cathode biofilms, Shannon indices increased from 4.3 (control) to 5.02 (100 μM Fe^2+^) and 5.54 (200 μM Fe^2+^), suggesting that Fe^2+^ affected microbial community structure of the electrode biofilms in MFCs. Principal component analysis based on OTUs showed three clusters, the anode biofilms of MFC without Fe^2+^ was separated from the anode biofilms of MFC supplemented with Fe^2+^ of 100 and 200 μM Fe^2+^ and the cathode biofilms (control, 100, and 200 μM Fe^2+^; **Figure 3**).

**Table 1:** Qualities of reads identified by Illumina Hiseq sequencing and bacterial diversity estimates based on OTUs (97% similarity).

Sample name	Effective tags	OTUs	Shannon	Chao1	Simpson	ACE	Good’s coverage
Anode (control)	53,807	824	5.21	908.307	0.884	900.018	0.997
Anode (100 μM)	53,136	630	3.716	691.84	0.733	703.657	0.998
Anode (200 μM)	54,932	679	4.706	785.135	0.886	796.327	0.997
Cathode (control)	51,054	692	4.3	755.5	0.748	773.924	0.997
Cathode (100 μM)	54,592	697	5.021	757.026	0.879	771.527	0.998
Cathode (200 μM)	50,373	741	5.542	810.327	0.927	813.045	0.997


### Bacterial Composition of the Anode and Cathode Biofilms

**FIGURE 4:**
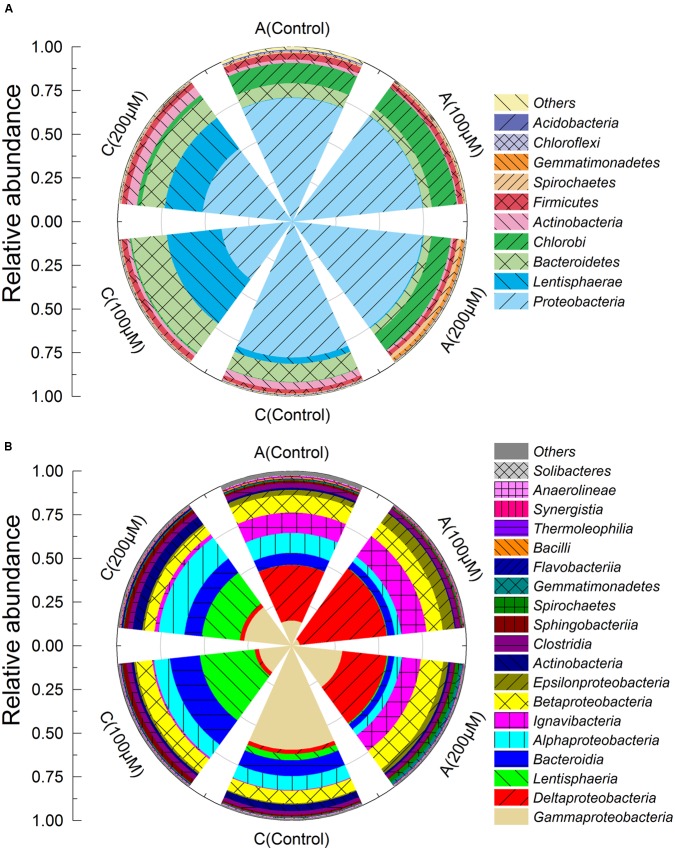
**Microbial community taxonomic wind-rose plots based on relative abundance of 16S rRNA sequences of the anode and cathode biofilms in MFCs at the phylum** **(A)** and class levels **(B)**.

The bacterial communities of the anode biofilms were substantially shifted when additional Fe^2+^ was supplemented in MFCs. *Proteobacteria* were the most dominant phylum observed both in the anode (71–75%, relative abundance) and cathode biofilms (41–78%) (**Figure 4A**). *Chlorobi* (11–14%) and *Bacteroidetes* (4–8%) were also predominant phyla in the anode biofilms. The relative abundances of *Lentisphaerae* in the cathode biofilms, were much higher than that in the anode biofilms, reached to 31% (100 μM Fe^2+^), 22% (200 μM Fe^2+^), and 4% (control). *Deltaproteobacteria*, *Ignavibacteria*, and *Betaproteobacteria* were the most predominant classes in the anode biofilms and accounted for 75% more or less, of which, the abundance of *Deltaproteobacteria* in the anode of MFCs with 100 μM reached to 50%, speculating that *Deltaproteobacteria* were the dominant class since MFC start-up period (**Figure 4B**). By contrast, microbial community structures of cathodes were different from anodes. *Alphaproteobacteria*, *Gammaproteobacteria*, *Bacteroidia*, and *Lentisphaeria* were the predominant classes on the cathodes. Cathodes of MFCs with additional Fe^2+^ had similar communities that were much different with control group.

**FIGURE 5:**
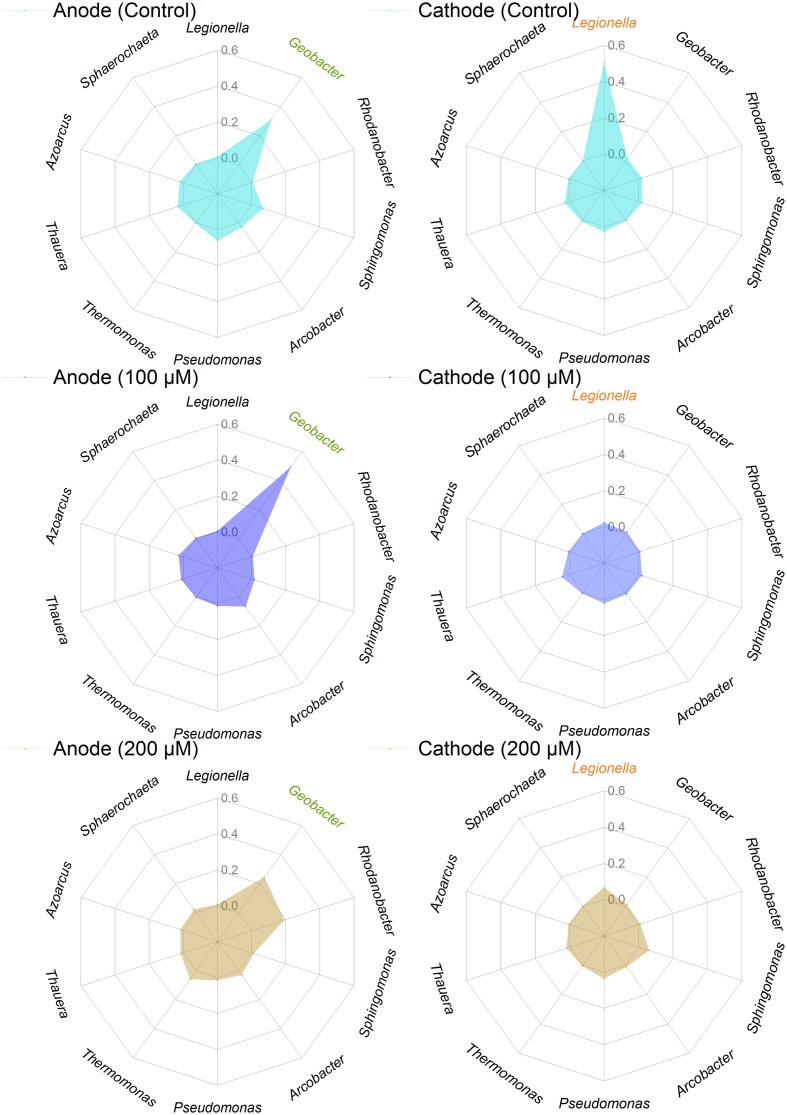
**Relative abundance of predominant genera in the anode and cathode biofilms in MFCs supplemented with different concentrations of ferrous iron**.

**FIGURE 6:**
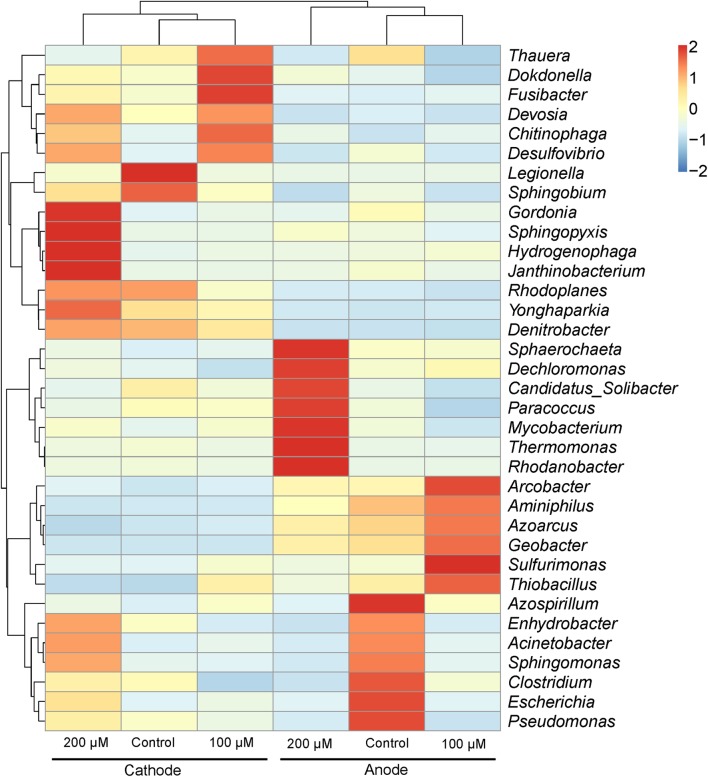
**Hierarchical cluster analysis of predominant populations in the anode and cathode biofilms in MFCs.** The genera with the relative abundance of the top 35 are shown. The species clustering tree is on the left and the sample clustering tree is on the top. Each box of the heatmap represents a *Z*-score, a positive score indicates a datum above the mean, while a negative score indicates a datum below the mean.

The predominant genera varied significantly among all anodes and cathodes biofilms (**Figure 5**). The majority of predominant populations in the control MFCs were affiliated with *Geobacter* spp. (30.7%) and *Legionella* spp. (50.3%). *Geobacter* was also the predominant genus in the anode of MFC supplemented with 100 and 200 μM Fe^2+^, the relative abundance of which population reached up to 49.3 and 24.4%. Another predominant genus in the anode biofilms of MFC (200 μM Fe^2+^) was affiliated to *Rhodanobacter* (19%). In the cathode biofilms of MFCs with 100 and 200 μM Fe^2+^, higher relative abundance of predominant genera belonged to *Legionella* spp. (2 and 6%), and no absolutely predominant populations were present. Hierarchical cluster analysis of microbial communities based on genus taxonomy revealed that the relative abundance of *Sphaerochaeta*, *Dechloromonas*, *Paracoccus*, *Thermomonas*, and *Rhodanobacter* increased in the anode biofilms of MFCs supplemented with 200 μM Fe^2+^ (**Figure 6**). Meanwhile, the relative abundance of *Gordonia*, *Sphingopyxis*, *Hydrogenophaga*, and *Janthinobacterium* in the cathode biofilms of MFCs with 200 μM Fe^2+^ were relatively higher than that in the cathodes biofilms of MFCs without Fe^2+^ and with 100 μM Fe^2+^, but higher proportion of *Thauera*, *Dokdonella*, *Fusibacter*, *Devosia*, and *Desulfovibrio* were observed in the cathode biofilms of MFCs with 100 μM Fe^2+^.

### Effect of Fe^2+^ on Predominant Populations in the Electrode Biofilms

Ferrous iron with appropriate concentration (100 μM) stimulated electrochemical activity of MFCs during the start-up period, but Fe^2+^ cannot enhance power output after 30 days of operation and higher concentration of Fe^2+^ had the negative effect (Wei et al., 2013), presumably the Fe^2+^ facilitated biofilm formation at the early stage. The metal ions may act as redox active sites in the enzymes which catalyze the electron transfer and redox reaction to affect the performance of bio-electrochemical systems (BESs) (Lu et al., 2015). In mature anode biofilms, pH decreased through different growth phases, showing that the pH is not always a limiting factor in a biofilm. Meanwhile, increasing redox potential at the biofilm electrode was associated only with the biofilm, demonstrating that microbial biofilms respire in a unique internal environment (Babauta et al., 2012). Oxidation of ferrous ion by microbes is an important component of iron geochemical cycle (Croal et al., 2004). Recent studies also confirmed that Fe^2+^ oxidation provides an energetic benefit for some microbes’ growth when using Fe^2+^ and acetate as the co-substrate (Muehe et al., 2009; Chakraborty et al., 2011). Illumina Hiseq sequencing of 16S rRNA gene indicated that Fe^2+^ shifted bacterial community and influenced enrichment of exoelectrogenic bacteria in the anode biofilms.

An excessive amount of metal salts may result in negative effects on the performance of BESs by inhibiting the activity of microorganisms (Jiang et al., 2011). The relative abundance of *Geobacter* increased from 30.7 to 49.3% in MFCs with 100 μM Fe^2+^ but decreased to 24.4% in MFCs with 200 μM Fe^2+^, implying higher Fe^2+^ concentration could not further enrich *Geobacter*. As a result, the power output of MFC with higher Fe^2+^ concentration (200 μM) was lower than control and 100 μM Fe^2+^ during MFC steady operation. *Rhodanobacter* accounted for a large proportion (19%) in MFCs with Fe^2+^ concentration of 200 μM. To date, the function of *Rhodanobacter* was mostly investigated on denitrifying (Green et al., 2012) and thiosulfate-oxidizing (Lee et al., 2007), but little is reported about Fe^2+^ oxidation especially mediated by C-type cytochromes (Croal et al., 2007; Bird et al., 2011). Whether it participates in interspecies interaction with *Geobacter* should be further proved. Other exoelectrogenic bacteria also formed a certain proportion in different anode biofilms, such as *Pseudomonas* (1–6%) and *Arcobacter* (3–7%) (Fedorovich et al., 2009; Yong et al., 2011). *Pseudomonas* has a positive role to benefit other exoelectrogens in anode biofilm under a high concentration of salt addition (Liu et al., 2016b). *Arcobacter* can be selectively enriched in an acetate-fed MFC and rapidly generates a strong electronegative potential (Fedorovich et al., 2009). It indicated that additional ions, like Fe^2+^, will take part in biofilm metabolism or microbial communication, which resulted in community structure changes.

The microbial communities on the cathodes clearly differed from the anodes biofilms in all MFCs. The most predominant genera in the cathode biofilms of MFCs without additional ferrous iron came from *Legionella* spp. (50.3% of relative abundance). However, the relative abundance of *Legionella* on the cathode biofilms declined to 2–6% with Fe^2+^ addition, suggesting that *Legionella* was inhibited by high concentration of Fe^2+^. The abundance of Fe(II)-oxidizing bacteria, *Janthinobacterium* (Geissler et al., 2011), in the cathode biofilms of MFC with 200 μM Fe^2+^ were relatively higher than other groups (**Figure 6**). Hierarchical cluster analysis based on genus taxonomy demonstrated that the response of predominant populations in the electrode biofilms to ferrous iron occurred, indicating the effect of ferrous iron on microbial community in MFCs.

### Effect of Environmental Factors on MFC Performances

Some environmental factors, such as nutrients, pH, and temperature, influence the performances of MFCs by changing microbial activity and community structure. Our study indicated that ferrous iron changed microbial community structures of electrode biofilms of MFCs. Other metals (e.g., Ca, Mg, Pt, Au, Pd, Fe, V, Mn) and metal-nanomaterials affected current generation of MECs by changing the metabolism and enzyme activity of microorganisms (Lu et al., 2015). These studies have analyzed effect of single metal on electricity generation by MFCs, however, the effect of combined metals on microbial community structure and performance of MFCs should be further investigated.

Neutral pH is considered as the optimal condition for current generation by MFCs (Gil et al., 2003; Jadhav and Ghangrekar, 2009). However, a higher pH has been demonstrated to enhance the electrochemical activity of riboflavin which is a metabolite responsible for extracellular electron transfer in some species (Yuan et al., 2011; Yong et al., 2013). By contrast, MFCs have also been operated at pH less than 4.0 and produced high current densities by acidophilic bacterium (Malki et al., 2008; Winfield et al., 2016). Previous studies proved that temperate substantially affected the performances of MECs or MFCs by shaping microbial community (Lu et al., 2011, 2012). Synergistic effect of metals, pH and temperature on performances of MECs and correlation analysis of these environmental factors should be further investigated in the future.

## Author Contributions

DX designed the experiment. QL performed specific experiments. QL, BL, and DX contributed to analyze the experiment data. QL, WL, WZ, XZ, and DX wrote the manuscript. All authors were involved in revision of the manuscript and approved its final version.

## Conflict of Interest Statement

The authors declare that the research was conducted in the absence of any commercial or financial relationships that could be construed as a potential conflict of interest.

## Footnotes

1 http://ccb.jhu.edu/software/FLASH/

2 http://drive5.com/uchime/uchime_download.html

3 http://greengenes.lbl.gov/cgi-bin/nph-index.cgi

4 http://sourceforge.net/projects/rdp-classifier/
